# The origin of heredity in protocells

**DOI:** 10.1098/rstb.2016.0419

**Published:** 2017-10-23

**Authors:** Timothy West, Victor Sojo, Andrew Pomiankowski, Nick Lane

**Affiliations:** 1 Department of Genetics, Evolution and Environment, University College London, Gower Street, London WC1E 6BT, UK; 2 Centre for Computation, Mathematics and Physics in the Life Sciences and Experimental Biology (CoMPLEX), University College London, Gower Street, London WC1E 6BT, UK; 3 Systems Biophysics, Faculty of Physics, Ludwig-Maximilian University of Munich, Amalienstr. 54, 80799 Munich, Germany

**Keywords:** composome, origin of life, protocell, RNA world

## Abstract

Here we develop a computational model that examines one of the first major biological innovations—the origin of heredity in simple protocells. The model assumes that the earliest protocells were autotrophic, producing organic matter from CO_2_ and H_2_. Carbon fixation was facilitated by geologically sustained proton gradients across fatty acid membranes, via iron–sulfur nanocrystals lodged within the membranes. Thermodynamic models suggest that organics formed this way should include amino acids and fatty acids. We assume that fatty acids partition to the membrane. Some hydrophobic amino acids chelate FeS nanocrystals, producing three positive feedbacks: (i) an increase in catalytic surface area; (ii) partitioning of FeS nanocrystals to the membrane; and (iii) a proton-motive active site for carbon fixing that mimics the enzyme Ech. These positive feedbacks enable the fastest-growing protocells to dominate the early ecosystem through a simple form of heredity. We propose that as new organics are produced inside the protocells, the localized high-energy environment is more likely to form ribonucleotides, linking RNA replication to its ability to drive protocell growth from the beginning. Our novel conceptualization sets out conditions under which protocell heredity and competition could arise, and points to where crucial experimental work is required.

This article is part of the themed issue ‘Process and pattern in innovations from cells to societies’.

## 1. Introduction

The origin of heredity is perhaps the first major innovation in biology. Most research has linked the emergence of heredity with the appearance of genetic replicators such as RNA [1–4]. The idea of an RNA world goes back to the late 1960s [5–7] and pleasingly solves the chicken-and-egg problem of which came first, DNA (which is mostly inert and cannot copy itself) or protein (which is catalytic, but has properties that are specified by DNA). Because RNA is capable of both catalysis and replication, it could theoretically have been central to the origins of heredity, and so life itself [5–7]. The fact that RNA remains the crucial intermediary between DNA and proteins reinforces this view. Plainly RNA was central to early evolution and the origin of the genetic code.

But there are some practical difficulties with the RNA-world hypothesis in its strongest form—the idea that ribozymes ‘invented’ metabolism as well as the genetic code. It has proved challenging to synthesize nucleotides via prebiotic chemistry [8–10]. The first successful synthesis of activated pyrimidine nucleotides was achieved as recently as 2009 [11], while purine nucleotides have yet to be produced by abiotic chemistry [10]. Even successful syntheses have required radically different conditions for separate reaction steps [10]. Nucleotide synthesis at the origin of life was presumably not facile. Even if synthesized at high concentration (or concentrated by eutectic freezing [12] or thermophoresis [13]), the polymerization of nucleotides to form RNA is equally challenging. Reports that cyclic nucleotides spontaneously polymerize in aqueous solution [14] have been difficult to replicate [15]. Wet–dry cycles in the presence of laminar beds of lipids can polymerize nucleotides into longer-chain RNA molecules [16], but the gap between wet–dry cycles and polymerization at high concentration in aqueous solution is great, with no obvious link between the two. Once RNA exists in solution (if provided with the polymerase enzymes needed for replication), selection is then almost invariably for replication speed rather than any form of coding or metabolism, giving rise to tiny, fast-replicating RNA sequences known as Spiegelman's monsters [17–19]. While thermal cycling can select for longer-chain RNAs [20], how that might promote coding and metabolism is not known. Competition for RNA replication speed alone typically leads to parasitic collapse [19]—evading such parasites (‘cheaters') is a pervasive theme in transitions to higher-level individuality (see [21] and [22]).

These difficulties could be resolved if RNA were initially formed at high concentrations inside self-replicating protocells, rather than free in solution. Specifically, protocells could in principle provide a structured, high-energy, catalytic environment capable of driving nucleotide synthesis via some form of energy coupling, perhaps involving acetyl phosphate derived from reactive thioesters [23–25]. Molecular crowding and phosphorylation in such confined, high-energy protocells could potentially promote the polymerization of nucleotides to form RNA [12,13,26]. The catalytic and coding properties of RNA formed within protocells would then be linked from the outset to the growth and proliferation of the protocells, rather than its own replication in free solution, potentially escaping parasitic collapse.

The ‘lipid-world’ hypothesis conjectures that the lipid composition of vesicle membranes could result in catalytic properties that generate further lipid precursors [27]. If some of these were incorporated into the membrane that generated them, the feedback would influence composition, leading to a form of rudimentary heredity [27–29]. The evolvability of such ‘composomes’ has been challenged as the replication fidelity is likely to be so low that fitter compositions could not be maintained by selection [30]. In addition, the supposed catalytic properties of a composome in generating its component precursors is described abstractly and does not obviously relate to known lipid catalytic properties, or to the broader metabolic biochemistry of cells. There is also no obvious path leading from limited lipid catalysis to mechanisms capable of generating other organics (e.g. amino acids, sugars, nucleotides) or to an RNA world encapsulated within proliferating protocells. Complex, self-amplifying chemical networks capable of self-replication have long been sought and would equate to a more robust form of heredity [8,31,32]. Plainly such heredity cannot depend on RNA, DNA, proteins, or even ‘proto’-nucleic acids with alternative sugars [33] or non-canonical nucleobases [34], which are also complex macromolecules, hence no more easily formed abiotically. But in the absence of enzymes or ribozymes, achieving the requisite degree of metabolic channelling has been described as the biggest hurdle at the origin of life [35].

Recent work on the early evolution of metabolism suggests a possible solution to this problem. The first cells were arguably autotrophs that grew from the reaction of H_2_ with CO_2_ via some form of the acetyl CoA pathway [23,36–40]. The ancestral form of this pathway might have been similar to that in modern methanogenic archaea [40], on the basis that methanogens use a membrane-bound NiFe hydrogenase (the energy-converting hydrogenase, Ech) to drive the reduction of ferredoxin [41], which in turn reduces CO_2_. This is important for two reasons: (i) Ech and ferredoxin are both iron–sulfur proteins with Fe(Ni)S cofactors that have lattice structures resembling FeS minerals such as greigite [36,42]; and (ii) Ech uses the proton-motive force to reduce ferredoxin [39–41]. Ferredoxin is capable of driving not only the first steps of CO_2_ fixation via the acetyl CoA pathway, but also the reverse incomplete Krebs cycle, arguably the hub of intermediary metabolism, from which fatty acids, amino acids and ultimately nucleotides are derived [23,43]. We have previously shown using computational simulations of proton flux that geochemically sustained proton gradients across the pores of hydrothermal vents can drive the operation of membrane proteins such as Ech without the need to actively pump protons out of cells [44]. This flux can be sustained if the cell membrane has a high proton permeability (equivalent to a fatty acid membrane), allowing protons trapped internally to leak out again across the membrane, given continuous alkaline hydrothermal flux [44]. In other words, cell growth could theoretically be powered by a single Fe(Ni)S membrane protein embedded in a fatty acid bilayer membrane in the presence of geochemical proton gradients.

Our previous study [44] assumed the existence of genes and proteins. The question we address here is how such a minimal genetically encoded system might have arisen. Specifically, could metabolic channelling across protocell membranes drive the evolution of a self-amplifying system capable of rudimentary heredity? We develop a computational model to examine the behaviour of fatty acid vesicles in the presence of FeS minerals and geologically sustained proton gradients. We show that simple physical interactions between FeS nanocrystals, hydrophobic amino acids and fatty acids generate positive feedbacks that drive protocell growth and reproduction, leading to a robust form of heredity at the level of the system. The protocells that are best able to generate organic matter inside themselves proliferate fastest, and should come to dominate the early ecosystem. While we do not specifically consider the synthesis of RNA, the model does show how membrane heredity could have preceded, and been an essential stepping stone to, an RNA world.

## 2. Model description

### (a) Model overview

**Figure 1.**
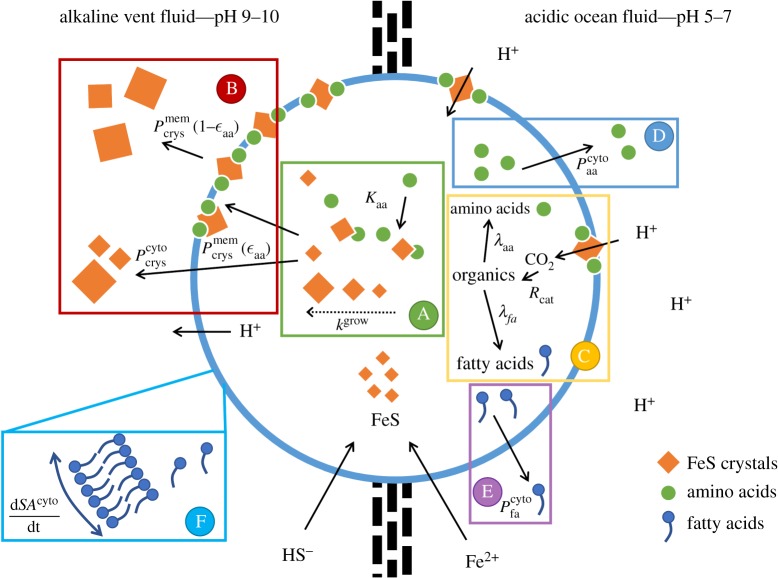
Model of FeS-catalyzed growth dynamics within a protocell. FeS nanocrystals spontaneously form from the reaction of Fe^2+^ from ocean waters and HS^–^ from hydrothermal fluids. (A) FeS nanocrystal growth and chelation by amino acids. (B) Crystal fluxes between the cytosol, membrane and external sink. Nanocrystal partitioning to the membrane depends on the presence of amino acids in the cytosol. (C) Amino acid-associated FeS nanocrystals embedded in the membrane (on the ocean side only) use the geological proton gradient to drive reduction of CO_2_ and formation of new organics inside the protocell. Amino acids (D) and fatty acids (E) are also subject to leak permeabilities towards the external sink. (F) Protocell growth is facilitated by the addition of newly generated lipids to the membrane, producing an increase in cell surface area. See Appendix A for more details.

We develop a computational model for the emergence of self-amplifying growth and reproduction in protocells. The dynamics follow from the interactions between amino acids and FeS minerals within simple vesicles bounded by fatty acid bilayer membranes. We assume that the vesicles are enclosed in the pores of alkaline hydrothermal vents, transected by geologically sustained proton gradients [23,40,45–49] (figure 1 gives a schematic representation). The model describes the evolution of the FeS crystal size distribution, determined by interactions with hydrophobic amino acids generated through catalysis by membrane-bound FeS crystals. The model involves three interlinked positive feedbacks: (i) chelation of FeS crystals by hydrophobic amino acids hinders the growth of crystals, increasing the proportion of smaller crystals and the surface area for catalysis; (ii) a proportion of FeS crystals partition to the membrane, with the rate of transfer from cytosol to membrane being enhanced for FeS crystals that are chelated; and (iii) the membrane-bound FeS crystals, when chelated by amino acids, resemble the active site of the proton-motive NiFe hydrogenase Ech and accordingly catalyze the reduction of CO_2_ to form new organic molecules within the protocells when in the presence of geochemical proton gradients. We consider the conditions under which these positive feedbacks could drive self-amplification and growth of protocells. Below we give a brief description of the model's dynamics. A fuller exposition of the system of ordinary differential equations that describes them is given in Appendix A.

### (b) Dynamics of crystal size changes

We describe the changes in crystal size in terms of the processes modified by interactions with amino acids in the protocell (figure 1, box A). The model does not explicitly consider the flow rates of Fe^2+^ or HS^–^ ions across the membrane to form FeS crystals, as these are unknown. Instead we assume that the two ions remain at steady-state concentrations across the vent–pore system. This assumption simply balances rates of loss with rates of gain, which should occur naturally in a diffusion gradient: if the rate of efflux increases then the gradient steepens, giving a linked increase in the rate of influx. For simplicity, we assume that the total volume of FeS crystals in the cytosol of protocells also remains at steady state, with the number of crystals at any given time being equal to this volume divided by the mean crystal size, equation (A 3); see Appendix A. We assume that size changes are proportional to the ratio between crystal growth and loss from the cytosol, so that if efflux is high (due to partitioning to the membrane or leaks to the external environment) there is a net decrease in mean cytosolic crystal size, equations (A 11) and (A 12). This is not caused by a change in the overall rate of crystallization of FeS; rather, the loss of FeS crystals from the cytosol means there is a lower likelihood of FeS crystallizing onto the surface of existing crystals, so fresh (smaller) crystals are more likely to be nucleated from aqueous Fe^2+^ and HS^−^. As a result, if the rate of crystal loss from the cytosol increases, mean crystal size within the protocell tends to decrease. Finally, the rate of crystal growth is directly hindered by the availability of cytosolic amino acids, so the total growth rate is slowed in a concentration-dependent manner, equations (A 5) and (A 10). We vary the strength of amino acid binding to FeS crystals so that a weak binding constant has little effect on crystal growth even when amino acids are present at high concentration, whereas a tight binding constant means that even low concentrations of amino acids can hinder crystal growth.

### (c) Partitioning of crystals to the membrane

Crystals are subject to three transport processes in the model, equations (A 6), (A 7) and (A 8): (i) a leak permeability of non-chelated crystals to the outside sink; (ii) partitioning of FeS crystals chelated by amino acids to the membrane; and (iii) a slow dissociation of membrane-bound crystals to the outside sink (figure 1, box B). The first process is a passive flux depending on the concentration difference between the inside and outside of protocells. We assume that the outside concentration is low (reflecting loss through hydrothermal flow), maintaining a continuous efflux of crystals from the cell. For simplicity, we do not assume an association between crystal size and the rate of loss—crystals of any size have an equal probability of being lost to the outside sink. The second flow involves the amino acid-dependent partitioning of crystals to the fatty acid membrane; again, we model this with a passive chemical flux, but this time modified by the cytosolic concentration of amino acids, so that higher concentrations of amino acids (or tighter amino acid binding) induce faster partitioning of FeS crystals to the membrane. Importantly, a higher rate of partitioning to the membrane equates to a higher rate of loss from the cytosol, hence a tendency to nucleate fresh, small FeS crystals in the cytosol. This corresponds to a decrease in mean cytosolic crystal size. Finally, membrane-bound crystals are subject to a rate of dissociation to the outside sink. This rate of dissociation is inversely proportional to the amino acid availability, so that when amino acid concentrations are high, loss from the system is low. We assume that chelated crystals are more likely to be hydrophobic, hence are more likely to remain in the membrane than non-chelated, less hydrophobic crystals.

### (d) Crystal catalysis of organic formation

The partitioning of FeS crystals chelated by hydrophobic amino acids to the membrane exposes the crystals to the geological proton-motive force, which we assume drives the reduction of CO_2_ to form new organic molecules inside the cell, equations (A 15) and (A 16), in a manner analogous to the membrane protein Ech (figure 1, box C). To simplify modelling, we condense the multifaceted dynamics of this proton-motive catalysis into a single parameter—the total molar rate of formation per unit area of catalyst. This gives a rate of organic synthesis that depends on the amount of membrane-bound crystal in the protocell and the catalytic turnover rate, which we vary in the model. We assume that the proton-motive FeS catalytic site lowers the initial endergonic barrier to CO_2_ reduction and that the product yield would ultimately reflect thermodynamic favourability. The synthesis of amino acids and fatty acids from H_2_ and CO_2_ is exergonic overall under alkaline hydrothermal conditions [50,51] and so should be favoured, whereas nucleotide synthesis is mildly endergonic under these conditions [50,51]. In the model, we assume that the fatty acids and amino acids produced would correspond in their relative proportions to the free-energy release predicted by thermodynamic modelling [50,51]. Amino acids and fatty acids are also subject to a leak permeability across the membrane, equation (A 17), (figure 1, boxes D and E).

### (e) Membrane growth and protocell division

The synthesis of new fatty acids via catalysis by membrane-bound FeS crystals is assumed to drive the growth of protocells through the addition of new fatty acids to the membrane (figure 1, box F). The growth in surface area is proportional to the number of new fatty acid molecules produced. We compute the increased protocell surface area by multiplying the number of new fatty acid molecules by the size of the carboxylic acid headgroup and dividing by two (for a bilayer), equation (A 18). We also consider protocell division and inheritance. These simulations assume a threshold point at which cells divide into two due to mechanical constraints of the bilayer and the cytosol. In practical terms, protocells divide when the membrane surface area has doubled. Daughter cells each receive half the amino acid-chelated membrane-bound FeS crystals, as well as cytosolic amino acids, fatty acids and FeS crystals. So cell division gives stable heredity, in which the self-amplifying system of amino acids, chelated FeS crystals and fatty acid membranes bearing chelated FeS crystals is passed onto the daughter cells.

## 3. Results

**Figure 2.**
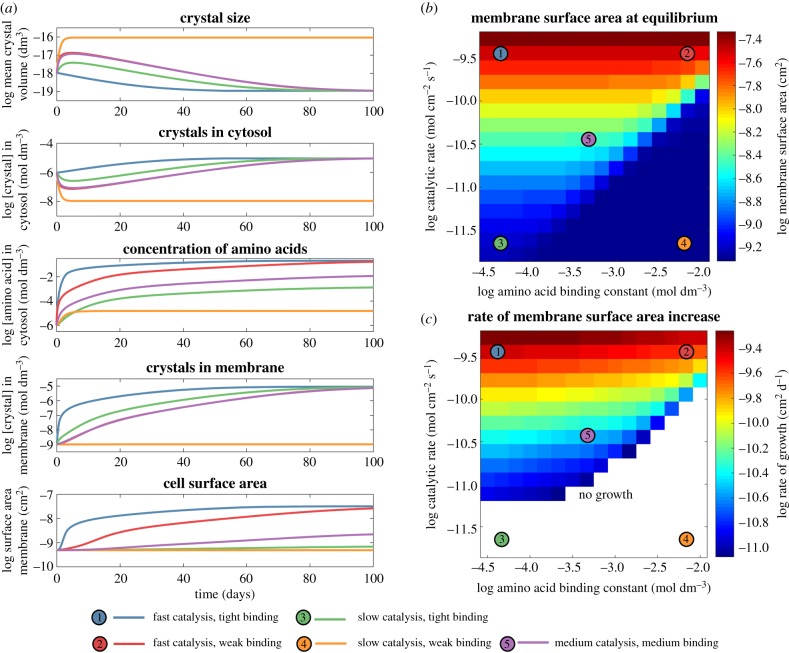
Parameters controlling protocell growth. The figure shows the effect of varying catalytic activity (*R*_cat_) and amino acid binding affinity (*K*_aa_) for FeS crystals. (*a*) Time courses for simulations computed for five parameter combinations corresponding to the five coloured sample points shown in (*b*) and (*c*). Parameters were chosen to demonstrate the dependence of crystal growth, production of organics, partitioning of FeS nanocrystals to the membrane, and growth in protocell surface area upon combinations of catalytic turnover rates and amino acid binding strengths. The correspondence of line colours to the parameter space are given in the bottom legend (see main text for more details). Parameter values: 1, fast catalysis (*R*_cat_ = 10^−9.4^ mol dm^–2^ s^–1^) tight binding (*K*_aa_ = 10^–4.3^ mol dm^3^); 2, fast catalysis (*R*_cat_ = 10^−9.4^ dm^–2^ s^–1^) weak binding (*K*_aa_ = 10^–2.2^ mol dm^–3^); 3, slow catalysis (*R*_cat_ = 10^−11.6^ mol dm^–2^ s^–1^) tight binding (*K*_aa_ = 10^−4.3^ mol dm^3^); 4, slow catalysis (*R*_cat_ = 10^−11.6^ mol dm^–2^ s^–1^) weak binding (*K*_aa_ = 10^−2.2^ mol dm^–3^); 5, medium catalysis (*R*_cat_ = 10^−10.4^ mol dm^–2^ s^–1^) medium binding (*K*_aa_ = 10^–3.7^ mol dm^–3^). (*b*) Parameter space representation of the protocell equilibrium surface area (cm^2^). Results demonstrate that the extent of cell growth is largely determined by the catalytic activity of the FeS crystals. (*c*) Parameter space representation of the rate of cell growth (cm^2^ day^−1^) during the growth period. Cases in which there was no growth are covered by the white section in the bottom half of the figure.

The results show that under certain parameter ranges, positive feedbacks can indeed drive protocell growth (figure 2). With tight amino acid binding (*K*_aa_ = 10^–4.3^ mol dm^–3^, figure 2*a* panel 1, curves 1 and 3), the mean crystal size falls relative to protocells with weak amino acid binding (*K*_aa_ = 10^–2.2^ mol dm^–3^, figure 2*a* panel 1, curves 2 and 4). Accordingly, the concentration of crystals in the cytosol increases in protocells with tight amino acid binding (*K*_aa_ = 10^–4.3^ mol dm^–3^, figure 2*a* panel 2, curves 1 and 3). The catalytic turnover rate (*R*_cat_) has little effect on crystal size or on the number of crystals in the cytosol, especially at high binding affinities. However, the cytosolic concentration of amino acids formed depends strongly on the catalytic rate. At higher catalytic rates, amino acids accumulate quickly in the cytosol (*R*_cat_ = 10^−9.4^ mol cm^–2^ s^–1^, figure 2*a* panel 3, curves 1 and 2). By contrast, in the case of tight amino acid binding (*K*_aa_ = 10^–4.3^ mol dm^–3^) but low catalytic rate (*R*_cat_ = 10^−11.6^ mol cm^–2^ s^–1^), the accumulation of amino acids in the cytosol is two orders of magnitude lower (figure 2*a* panel 3, curve 3).

Chelation by amino acids promotes the partitioning of FeS crystals from the cytosol to the membrane (figure 2*a* panel 4). The rate of partitioning to the membrane depends mainly on the tightness of amino acid binding, with tight binding (*K*_aa_ = 10^–4.3^ mol dm^–3^, figure 2*a* panel 4, curves 1 and 3) promoting rapid transfer of FeS crystals to the membrane. Weak binding can be compensated by faster catalytic rates (*R*_cat_ = 10^−9.4^ mol cm^–2^ s^–1^; figure 2*a* panel 4, curve 2, or *R*_cat_ = 10^−10.4^ mol cm^–2^ s^–1^, panel 4, curve 5) as this produces more amino acids. Only when both binding affinity and catalytic rate are low (figure 2*a* panel 4, curve 4) do FeS crystals fail to accumulate in the membrane.

The protocell surface area reflects both binding affinity and catalytic rate (figure 2*a* panel 5). Here, the binding affinity mainly affects the speed of growth, with tight binding (*K*_aa_ = 10^–4.3^ mol dm^–3^, figure 2*a* panel 5, curve 1) promoting faster growth than weaker binding (*K*_aa_ = 10^–2.2^ mol dm^–3^, curve 2) but ultimately the same equilibrium surface area is reached. In contrast, lower binding affinities and slower catalytic rates produce limited growth (figure 2*a* panel 5, curve 5) or no growth at all (figure 2*a* panel 5, curves 3 and 4). In all cases, the curves eventually reach equilibrium; as the surface area increases, the rate of loss of crystals, amino acids and fatty acids eventually balances their rate of formation.

**Figure 3.**
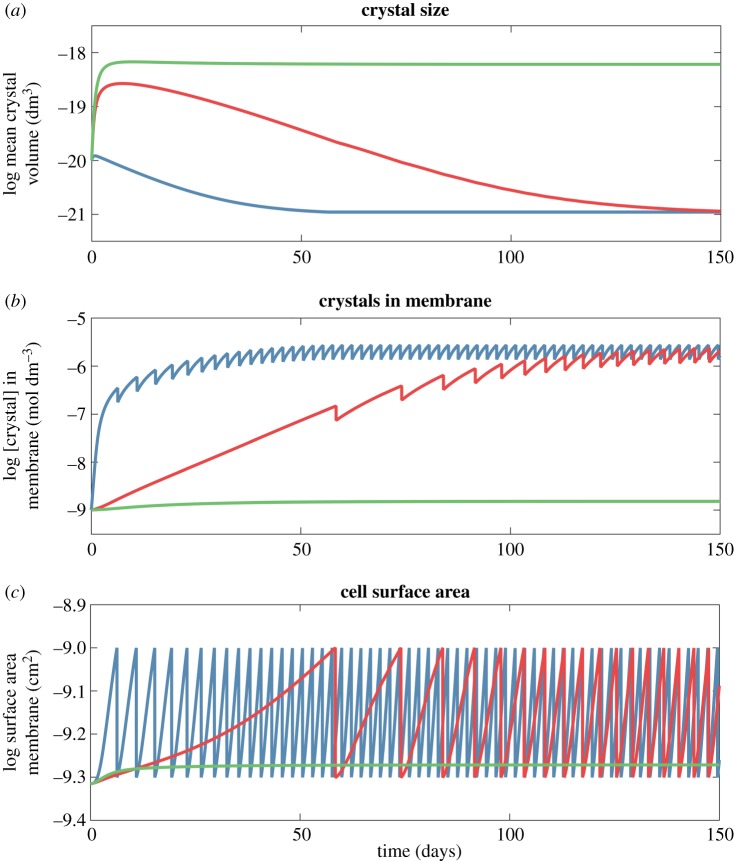
Protocell division as a function of amino acid binding. At a threshold cell surface area of 10^–9^ cm^2^, the cell divides and cytosol constituents are reorganized. The rate of growth depends on the strength of amino acid binding (*K*_aa_). The blue curve shows tight binding (*K*_aa_ = 10^–4.3^ mol dm^–3^), the red curve shows weaker binding (*K*_aa_ = 10^–2.7^ mol dm^–3^) and the green curve shows the weakest binding considered (*K*_aa_ = 10^–2^ mol dm^–3^). (*a*) Time course of crystal volume evolution. So long as intracellular amino acids are conserved across a protocell division, FeS nanocrystal chelation can continue. (*b*) Dynamics of membrane-bound crystal concentration demonstrates that a loss of half of membrane crystals during division is not sufficient to significantly slow cell growth. (*c*) Protocell surface area dynamics indicating growth and division in the case of the two tightest binding coefficients (blue and red curves) compared with no growth (green). Protocell division intervals decrease until the catalytic activity of the cell reaches equilibrium. The turnover rate was held constant for all three simulations (*R*_cat_ = 10^−10.4^ mol dm^−2^ s^−1^).

The equilibrium surface area depends mainly on the catalytic rate of FeS nanocrystals in the membrane (figure 2*b*, point 1 versus point 3; point 2 versus point 4), whereas the amino acid binding constant has relatively little effect (figure 2*b*, point 1 versus point 2). In contrast, the rate of protocell growth depends on the amino acid binding constant as well as the catalytic rate (figure 2*c*). A tight binding constant (figure 2*c*, point 1, *K*_aa_ = 10^–4.3^ mol dm^–3^) results in faster partitioning of FeS crystals to the membrane compared with a lower binding constant (figure 2*c*, point 2, *K*_aa_ = 10^–2.2^ mol dm^–3^), and a three-fold faster rate of growth. This can be seen more clearly in figure 3, which depicts the growth and division of protocells as a function of amino acid binding. Tight binding (blue line, *K*_aa_ = 10^–4.3^ mol dm^–3^) results in a faster reduction in crystal size (figure 3*a*), more rapid partitioning of crystals to the membrane (figure 3*b*), and fast growth and division of protocells (figure 3*c*). The process is similar but slower if the strength of amino acid binding is two orders of magnitude lower (red line, *K*_aa_ = 10^–2.6^ mol dm^–3^), but there is no growth at all if amino acid binding falls by approximately another order of magnitude (green line, *K*_aa_ = 10^–2^ mol dm^–3^). The periodicity indicates some loss of FeS crystals from the membrane during cell division, but this interferes little with growth (figure 3*b,c*). We assume that cells divide when they attain a surface area of 10^–9^ cm^2^, which restores the original cell surface area and volume, driving sustained growth.

## 4. Discussion

The model shows that a rudimentary form of heredity can drive the synthesis of organic molecules within protocells, promoting their growth and reproduction. The model is based on a very simple form of carbon fixation in cells, the use of the proton-motive force to drive the reduction of CO_2_ by H_2_ in some autotrophic organisms. This conception is based on the membrane-bound proton-motive NiFe hydrogenase Ech (energy-converting hydrogenase) found in contemporary methanogens [39–41]. Unlike other forms of CO_2_ reduction such as photosynthesis, which involve multiple enzyme complexes, CO_2_ fixation via Ech requires only two Fe(Ni)S enzymes: Ech itself and ferredoxin [39]. The Fe(Ni)S cofactors in these proteins have similar structures to the FeS minerals that form spontaneously in many hydrothermal systems [36,42]. The geological process of serpentinization generates H_2_ at mM concentrations in alkaline hydrothermal systems [49]. CO_2_ concentrations in the oceans were probably orders of magnitude higher 4 billion years ago than today [52,53], hence Hadean oceans were more acidic and alkaline vents were less carbon starved than modern systems. Alkaline hydrothermal flow sustains steep pH gradients of 3–5 pH units within the porous interior of these vents, with alkaline fluids at a pH of around 11 [46–49] and percolating Hadean ocean waters at perhaps pH 6. [52,53]. This means that all the required conditions for the abiotic equivalent of CO_2_ reduction via Ech should have been present in Hadean alkaline hydrothermal vents: high concentrations of H_2_ and CO_2_, a geochemically sustained proton-motive force, and Fe(Ni)S minerals equivalent in structure to the active sites of modern FeS proteins such as Ech and ferredoxin [23,36–41]. These conditions should drive CO_2_ reduction across fatty acid membranes to form new organics within protocells, promoting the emergence of faster growing protocells, giving rise to a rudimentary form of heredity.

The emergence of heredity in abiotic protocells in our model is driven by several interlinked positive feedbacks, depicted in figure 1, which together give rise to self-amplifying growth. FeS minerals chelate simple organic molecules such as hydrophobic amino acids, which we assume can influence three properties of FeS nanocrystals: (i) their growth rate, (ii) partitioning to lipid bilayers, and (iii) catalytic capability. First, we assume that the chelation of hydrophobic amino acids to the surface of growing FeS crystals will hinder their growth, blocking further attachment of Fe^2+^ and HS^–^ to a crystal surface. A larger number of small nanocrystals should therefore form from the same total FeS input, giving a greater catalytic surface area. Second, chelation with hydrophobic amino acids (or other small organics) should promote the partitioning of nanocrystals to the lipid phase of protocell membranes, in part because chelated nanocrystals are more hydrophobic, and in part because small nanocrystals are more likely to be physically accommodated within the lipid bilayer (but see below). Third, we assume that the chelated FeS crystals have improved catalytic properties relative to naked FeS crystals, because (in addition to the larger total surface area) the physical interactions between FeS nanocrystals and amino acids will tend to mimic the active site of FeS proteins such as Ech and ferredoxin. Critically, partitioning of chelated FeS minerals to the membranes of a protocell situated in a geologically sustained proton gradient would provide a simple abiotic equivalent to Ech in methanogens, which draws on the proton-motive force to drive CO_2_ reduction by H_2_ [39–41].

From this model, we determined the parameter space where protocell growth and reproduction can occur (figure 2). The results show that, across a range of values, growth and heredity are possible in principle. At higher catalytic rates and tighter amino acid binding constants, protocells grow and divide (figure 3), corresponding to the three criteria for innovations laid out by Hochberg *et al*. [54]—a gain in phenotypic complexity (chelated FeS crystals partitioning to the membrane); a novel function (autotrophic growth of protocells); and a significant ecosystem impact (rudimentary heredity enabling the fastest replicators to dominate). Little is known about whether the specific parameter values used in our modelling are reasonable. They should not be interpreted literally, but thought of as indicative of conditions that promote protocell functioning. The advantage of rigorous modelling is that it dissects the individual steps involved and points to what needs to be tested experimentally, which should help determine the plausibility of the conceptualization.

Nonetheless, several considerations suggest that the model parameters are not unreasonable. FeS minerals can catalyze CO_2_ reduction to form organics, albeit the reduction is not facile and most researchers have considered CO rather than CO_2_ [55–58]. Heinen & Lauwers [58] showed that CO_2_ can be reduced to various organics by H_2_S. Applying an electrochemical overpotential of 1 V can also drive CO_2_ reduction to pyruvate on Fe(Ni)S nanoparticles [59,60]. The pH-dependent modulation of reduction potential suggested here (equivalent to the mechanism of Ech in methanogens [40,61]) can drive formation of formaldehyde at nM [61] or even µM quantities (N Lane 2016, unpublished observations). Theoretical thermodynamic modelling shows that the synthesis of total cell biomass, and especially amino acids and fatty acids, is favoured from H_2_ and CO_2_ under alkaline hydrothermal conditions (pH 11, 25–125°C) [50,51]. We have delineated a testable succession of carbonylation and hydrogenation reactions by which pH differences across FeS barriers could drive the reverse incomplete Krebs cycle [62]. The postulated first step, Fe(Ni)S catalysis of organic synthesis from H_2_ and CO_2_, is therefore plausible.

Likewise, evidence suggests that amino acids such as cysteine can interact with FeS minerals, altering their catalytic properties and surface area [63,64]. α-Ketoacids such as pyruvate (and even simpler organics such as formaldehyde, CH_2_O) can promote the formation of greigite (Fe_3_S_4_) rather than iron pyrites (FeS_2_) from the metastable FeS mineral mackinawite (FeS) [65]. This is noteworthy because the Fe_3_S_4_ clusters found in ferredoxin, Ech and other FeS proteins have the same unit-cell structure as greigite [36,42]. So the basic premise that organic interactions with FeS minerals can modulate their catalytic properties also has some foundation. Whether chelation by organics can obstruct crystal growth is uncertain, but can be addressed experimentally. Likewise, little is known about whether chelated FeS minerals can partition to lipid bilayers. FeS minerals such as mackinawite do have hydrophobic surfaces and can adsorb lipids and other hydrophobic molecules [64–67], so the premise is not unreasonable, but experimental elucidation is needed. A more complicated rendering of the model could investigate changes in crystal mass over time, but the outcomes would most likely be trivial. If crystals were to grow so large that they occluded the entire cytosol, or burst the membrane when lost from the cell, then plainly those protocells would not be capable of hereditary proliferation. Conversely, if the rate of crystal growth or nucleation fell so low that the crystal load dwindled, then growth would cease. We therefore consider only the conditions where growth and cell division are possible, and specify the parameter range in which heredity could in principle be established. That gives specific predictions about crystal growth and membrane partitioning that can be tested experimentally.

Another issue that needs to be addressed experimentally concerns the distance over which geological proton gradients can operate. The model assumes that steep pH gradients of 3–6 pH units operate across micrometre distances. Microfluidic studies show that laminar flow through hydrothermal-scale pores can support pH gradients of up to 6 pH units across micrometre distances, so steep gradients are certainly feasible [68]. However, the specific topology of the model is abstract, and might represent too generous a scenario. More realistically, we envisage laminar flow through remote channels, linked by proton-permeable inorganic barriers or through fluid connections to static channels cut off from the main hydrothermal flux [69]. The membranes of protocells growing in such static channels could then act as the principal proton insulation between remote active channels, enabling steep pH gradients to form even though they are supported by distant hydrothermal flow. This hypothesis needs to be tested experimentally.

The system of membrane heredity described here is both simpler and more closely based on living cells than earlier proposals. Cavalier-Smith has invoked a form of membrane heredity in ‘obcells’ in which a hydrophobic genetic machinery is inserted into the membrane of an inverted cell, giving fixed units of selection (arguably avoiding the problem of Spiegelman's monsters) [70,71]. But this system presupposes the existence of peptidyl-tRNAs with hydrophobic tails associated with obcell membranes, and growth on polyphosphate surfaces [70,71]. There is no evidence for the existence of such obcells or for growth coupled non-enzymatically to polyphosphate surfaces. Perhaps the closest equivalent to our proposal was from Morowitz *et al*. in 1987 [72]. They presented a strong argument for protocells as the optimal context for nurturing the origins of RNA and heredity, though their proposed basis for protocell growth was rather different to ours. They suggested that protocells acquired rare amphiphilic pigments from the environment, which enabled them to conserve light energy as a proton-motive force across membranes, driving the formation of pyrophosphate and the coupled conversion of more common environmental precursor molecules into new membrane amphiphiles [72]. The idea is similar to ours as it involves proton gradients across protocell membranes. However, there is no heredity because the growth of new protocells depends on random interactions with pigments and amphiphile precursors in the environment—daughter cells have no better likelihood of making copies of themselves than any other protocells in the same environment. Some specific details are also problematic. The active generation of proton gradients across leaky fatty acid membranes is not simple [44] and the idea calls on a rudimentary form of photosynthesis at the origin of life, for which there is no evidence from either phylogenetics [43,73–75] or metabolic physiology [43,76–78].

In our view, the presence of some form of self-amplifying heredity is a crucial step in the origin of living systems, and must have preceded the replicative heredity of nucleotides. This is also the pretext of the ‘lipid-world’ hypothesis [27–29], which developed the ideas originally laid out in Morowitz *et al*. [72] to give a rudimentary membrane heredity in which the lipid composition of vesicles (the ‘composome’) can be influenced through lipid catalysis, so that environmentally produced amphiphiles are assembled into the membrane with similar composition to the parent vesicle [27–29]. While the GARD (graded autocatalysis replication domain) model shows that inheritance is possible in principle [28,29], the idea has been challenged on the basis that stochastic variations prevent robust inheritance and limit evolvability [30]. There are several other limitations too. The model depends on feeding with amphiphiles from the environment, and so can only evolve if amphiphiles with the right properties happen to be present. Such heterotrophic origins are inconsistent with phylometabolic evidence suggesting that the first cells were autotrophic, growing from H_2_ and CO_2_ [40,42,73–78]. Perhaps more importantly, there is no obvious link between the lipid-world hypothesis and the origins of catalysis by amino acids, short polypeptides, nucleotides or ribozymes—no clear path from a lipid world to the origins of RNA and genetic heredity.

In contrast, our proposal generates true heredity, in which faster-replicating protocells are more likely to make copies of themselves, through a series of simple positive feedbacks. These lead to the self-amplifying synthesis of new organic matter, including both amino acids and fatty acids, inside the protocell itself. Internal synthesis provides a natural concentrating mechanism as well as a localized high-energy environment capable of promoting further chemistry, including the eventual synthesis of nucleotides, RNA and DNA. This is consistent with autotrophic origins and with the specific mechanism of carbon fixation in methanogens [23,36–41]. Rather than being linked specifically with energy transduction (via pyrophosphate or ATP), the proton gradient is coupled to carbon fixation and so drives protocell growth directly. We are not calling on complex molecules such as pigments from the environment, merely Fe^2+^ from the ocean and HS^–^ from hydrothermal fluids. The form of heredity described here operates at the level of the system as a whole, which is conceptually similar to Ganti's chemoton model [79] in that it links metabolic channelling (through a membrane-bound proto-Ech in our case) with the intra-protocellular synthesis of amino acids and fatty acids, driving growth. The protocells best able to make copies of themselves pass on these properties at the system level: more cytosolic amino acids, more chelated FeS nanocrystals, more partitioning of these crystals to the membrane phase, more organic synthesis and faster growth.

In sum, the computational model developed here shows that a simple form of heredity, based on positive feedbacks in the chelation and partitioning of FeS nanocrystals to the membrane, could drive the growth and proliferation of protocells under geologically sustained proton gradients. The fastest-growing protocells are more likely to generate higher concentrations of organics internally, which should promote more complex biochemistry and energy coupling, ultimately giving rise to genetic heredity—an RNA world linked to the growth and proliferation of protocells.

## Supplementary Material

Supplementary Table 1

## Data accessibility

This article has no additional data.

## Appendix A. Extended description of the computational model

### (a) General model description

The protocell model presented comprises three compartments: a spherical cytosol; a membrane of fixed thickness equivalent to the fatty acid bilayer which encapsulates the cytosol; and an external sink of infinite volume. We model the growth of crystals within the cytosol by assuming that crystals grow with a constant rate over time, and that losses from the system (due to membrane association or passive diffusion) are replaced by instantaneous nucleations that arise from an equilibrium of Fe^2+^ from the ocean water and HS^–^ from the alkaline vent. The rate of crystal growth is modified by the availability of amino acids that are assumed to chelate crystals and hinder their growth. Chelated crystals are assumed to have a higher propensity for partitioning to the hydrophobic membrane and so membrane association will increase with the availability of amino acids. The proximity of membrane-associated crystals to the proton gradient that exists across the cell membrane is thought to facilitate catalysis and so we use a rate of organic production that is dependent on the total surface area of membrane-bound FeS crystals. A fraction of the total organic yield gives rise to new amino acids and fatty acids in the system. The hydrophobic fraction of amino acids can chelate FeS crystals, whereas fatty acids contribute to growth of the cell membrane. As the membrane surface area grows, it eventually reaches a critical point at which the cell divides and the membrane-bound crystals are divided between the cell and its progeny.

### (b) Model notation, terminology and units

Volumes, surface areas, radii, numbers of molecules and concentrations are given by , , *r*^C^,  and [*x*]^C^, respectively, where the subscript *x* indicates the species and *C* is the compartment to which it corresponds. If the parameter describes the geometry of a compartment, then the species is omitted (e.g. the volume of the cytosol is given by ). All units of length are standardized to use cm as a base unit for geometries (except in the case of concentrations, in which dm is more conveniently used), and s for time. Species used are: amino acids (aa), fatty acids (fa), FeS crystals (crys), organic molecules (orgs) and carbon dioxide (CO_2_). The three compartments in the model are designated by the superscripts  , for the cytosol, membrane and external sink, respectively. We use *k^z^* to indicate rate constants for process *z* (s^−1^ assuming first-order kinetics); *K_x_* to indicate saturation constants for species *x* (mol dm^−3^);  for the molar turnover of process *z* (mol cm^−2^ s^−1^);  for permeability coefficients of species *x* diffusing through the interface of *C* (cm s^−1^); *v_x_* for molar rates of formation of species *x* (mol dm^−3^ s^−1^); *λ_x_* is the fraction of total organic formation that yields species *x* (unitless); and *A*_N_ is Avogadro's number (mol^−1^). The number of molecules *n* is referred to at points in the formulation, in these cases . In the case of crystal population dynamics, the overbar (e.g. ) indicates the mean value, and bold parameters (e.g. ***V***) are used to indicate that the value is the sum over the whole population (i.e. ).

### (c) Passive chemical diffusion

Chemical diffusion of uncharged species *x* across the interface of compartment C_1_ is modelled using the following generic equation:

A 1

The flow is dependent on *P*, the permeability coefficient; *SA*/*V*, the diffusion surface area/volume ratio; and Δ*C*, the chemical driving force between compartments. Fluxes in the model are assumed to occur as single-step processes between cytosol and sink, cytosol and membrane or membrane and sink.

### (d) Compartments and geometry

The protocell cytosol (cyto), membrane (mem) and external environment (sink) are modelled using two finite volume compartments (cytosol and membrane) and a sink of infinite volume. The cytosol is modelled as a sphere of fixed volume. It is enclosed by the membrane compartment which has a volume *V*^mem^ equal to the enveloping spherical shell:

A 2

where  and  are the radii of the cytoplasm and membrane, respectively. We compute the membrane volume to impose a diffusive limit on the maximum concentration of FeS crystals that can move into it from the cytosol. For simplicity in the model we do not account for water movement and so we fix the cytosolic volume throughout simulations. Instead, the surface area is dynamic and subject to a growth proportional to the amount of fatty acid that is added to the membrane compartment. The thickness of the membrane is constant, but its surface area could grow without an increase in internal volume through furrowing or invaginations of the membrane. We assume that the internal surface of the membrane is equal to the surface area of the cytoplasm .

### (e) Initializing the crystal population

To initiate the simulation, we set an initial mean crystal size,  and assume that the cytoplasm has a fixed total volume of crystal,. From this, the number of cytosolic crystals is:

A 3

At each integration step the number of crystals within the cell is recalculated given that the mean crystal size can vary through time (see below). A more useful expression is the crystal concentration given the volume of the cytosolic compartment:

A 4

where *A*_N_ is Avogadro's number.

### (f) Amino acid binding to FeS crystals

Several processes in the model, such as crystal growth and membrane association, are dependent on the availability of interacting amino acids within the cytosol. We model amino acid binding to FeS crystals using a saturating Michaelis–Menten expression, with  indicating the fraction of crystals bound to amino acids:

A 5

The saturation constant *K*_aa_ sets the concentration of amino acids at which there is half maximal binding. Smaller values of the binding constant are indicative of a higher affinity of amino acids for crystals. We use  several times in the model to scale rates of crystal growth as well as diffusion of crystals into and away from the membrane. This allows modelling of amino acid-dependent rates of crystal chelation and membrane association.

### (g) Crystal movement and membrane partitioning

Crystals are subject to diffusion between compartments, which is modelled using the general flux equation (A 1). Diffusion of unbound crystals between the cytosol and sink is given by:

A 6

where  is the permeability constant of crystal crossing the membrane. Diffusion of amino acid-bound crystals between the cytosol and membrane is given by:

A 7

The diffusion term here is multiplied by the amino acid-dependent modifier , such that when , the potential rate of diffusion is near its maximum (see equation (A 5)).  is the permeability constant of crystal movement across the interface between cytosol and membrane. Finally, diffusion of amino acid-bound crystals between the membrane and sink is given by:

A 8

where again, modification by the term  creates an inverse dependence on the availability of amino acids. Thus, the potential for crystals to diffuse away from the membrane is minimized when .

The total rate of change of concentration of the membrane-bound crystals is equal to the sum of the fluxes:

A 9

### (h) Crystal growth rates

In the model, we restrict our description of crystal growth to the evolution of the mean of crystal size distribution. For simplicity, we assume that the total volume of crystal within the cell is fixed and the total availability (i.e. number of crystals) is proportional to the total volume of crystal divided by the mean crystal size within the cytosol, equation (A 3). Crystal growth arises from the deposition of FeS on the crystal surface. We consider the rate of growth as a constant process of diffusion that is modified by the crystal concentration and surface area*.* When the concentration is high we assume that deposition is high. We account for the impact of crystal movement on the crystal size distribution by assuming that loss of crystals from the system has a negative effect on growth (i.e. high efflux results in crystal shrinkage). Losses in crystal mass due to membrane association or permeation are assumed to be replaced by instantaneous re-equilibration with aqueous Fe^2+^ and HS^−^ ions that are maintained at a constant ambient concentration within the cytosol. Thus, the overall change in the mean crystal size decreases when crystal deposition is smaller than crystal efflux due to a net seeding of new crystals within the cell (compensating for the loss of mass from the system), and newly seeded crystals push the population mean to smaller values.

To calculate the rate of change in mean crystal size, we first compute an estimate of the rate of change that results from deposition at the crystal surface. We estimate the total molar change to the crystal population by assuming that the process can be modelled as diffusion across the surface area of the population with a driving force dependent on the distance of crystal concentration from its saturation point *K*_crys_:

A 10

where  is a permeability constant that encapsulates the rate of diffusion across the interface of the crystal surface (surf), and  is the total surface area of crystal in the cytosol. *K*_crys_ sets the driving force of the diffusion process of crystal. This constant effectively slows growth of the population as  decreases. This assumes that growth is slowed in larger crystals (for discussion of this effect see [80]). The entire flow is modulated by the amino acid/crystal chelation factor  such that when , the potential rate of crystal growth is hindered due to the chelating action of amino acids, equation (A 5). For similar formulations treating crystal nucleation/growth as a diffusive process see [80,81].

### (i) Crystal volume dynamics

We then make use of the computed flows to modulate rates of change of the mean crystal size. In the model, we assume that crystal growth occurs at a rate that is proportional to the difference between the minimum crystal size (at nucleation) and the mean population crystal size. Furthermore, the entire process is modulated by the balance between the total gains and losses of crystal mass in the system that arise from the transport and growth processes described in the previous sections.

We first compute the total crystal mass gains and losses by, respectively, summing the positive and negative flows involving the cytosolic compartment:

A 11

where the plus or minus signs indicate the absolute value of the positive or negative components of the rate of crystal concentration change. We then use the ratio of the gains to losses to determine the evolution of the crystal size distribution. The total rate of change for the mean of the crystal size distribution of the population is given by the following equation:

A 12

where *k*^grow^ is a rate constant that determines the evolution of the mean size of the crystal population, and  sets a bound on the minimum crystal size, such that when  approaches , the total rate of growth is slowed due to the decreased surface area available for diffusion. The equation is multiplied by the ratio of gain to losses from the system, so that when  mean crystal size increases, due to a net accumulation of mass on crystal surfaces, while when  mean crystal size decreases, as losses of crystal from the system are compensated by nucleation of new, smaller crystals. When *gain* *≈* *loss* then there is no net change in mean crystal size, as the formation of new crystals is balanced by the loss of older (larger) crystals from the system. The minimum crystal size set by  and the effective limit on the growth of population imposed by *K*_crys_ in equation (A 10) ensures that the range of possible crystal size is bounded.

### (j) Catalytic action of membrane-bound FeS crystals

In the model, reduction of CO_2_ to form organics is catalyzed by membrane-bound FeS crystals utilizing the chemiosmotic proton gradient that exists across the membrane. We do not explicitly model the proton gradient but instead absorb its effects upon catalysis into the parameter  which describes the rate of organic production per unit surface area of available catalyst (in the form of membrane-associated FeS crystals). For simplicity we assume that changes in size of the crystals in the cytosol are instantaneously translated to those found in the membrane. That is .

The crystal surface area (i.e. the site of catalytic action) is computed as the surface of a cuboid with a volume given by the average of the crystal size distribution:

A 13

so the total surface area of membrane-bound crystal is given by:

A 14

Hence the maximum molar rate of product formation is given by:

A 15

Adsorbance of CO_2_ onto the FeS catalyst is modelled using Michaelis–Menten kinetics, whereby adsorbance rates saturate depending on the affinity of the ligand for the binding sites of the crystal. Thus, the total CO_2_-dependent rate of organic formation is given by:

A 16

We assume the concentration of CO_2_ to be ten times higher than that at the Lost City hydrothermal vents today [78], which is conservative given that CO_2_ levels were probably several orders of magnitude higher in the Hadean [48,49]. Following thermodynamic modelling, new organics are most likely to include fatty acids and amino acids [46,47]. A quarter of the total organic output is assumed to be fatty acid and is partitioned to the bilayer. One tenth of the total organic output is assumed to be hydrophobic amino acids that chelate FeS crystals. We assume that all organics are formed on the inside surface of the membrane and contribute to concentration changes in the cytosolic compartment. The individual rate of change of cytosolic concentrations of amino acids and fatty acids are, therefore, computed as fractions of the total rate of production of organics with the addition of a leak permeability between the cytosol and sink ( for amino acids and fatty acids, respectively):

A 17

The terms *λ*_aa_ and *λ*_fa_ are the relative fractions of the total yield for hydrophobic amino acids and fatty acids (i.e. 1/10 and 1/4, respectively).

### (k) Membrane growth

We assume membrane growth is proportional to the rate of fatty acid production, and that fatty acids are instantaneously partitioned to the membrane bilayer. The rate of protocell surface area change is then:

A 18

where the changes in cytosolic fatty acid concentration are converted to a change in membrane surface area. The molar rate is first converted to a number of molecules via Avogadro's number (*A*_N_), and then the total surface area of the fatty acid head groups is computed by multiplication with , the single fatty acid head group size. This total area change due to fatty acid addition to the membrane is divided by 2 in order to give an approximation to the surface area of the inner face of the bilayer.

Since the model describes cell growth as a rate of surface area change of the cytosol, we re-compute the volume of the membrane at each time step using the radius of the cytoplasm given by the new surface area *SA*^cyto^:

A 19

### (l) State equations

Following the rationale outlined above, the dynamics of the system are governed by five state variables that are integrated over time:

A 20

### (m) Protocell division

Simulations of cell division assume a critical threshold at which the cell divides due to constraints of the cell mechanics. At this threshold, we assume that the mean of the crystal size distribution is inherited by progenitor cells and is unchanged. Furthermore, the concentration of amino acids and the concentration of membrane-bound crystals is divided by two as the cells become diluted by water movement during division. We also assume partitioning of cytosolic fatty acids in the process of division, so they are reduced to half their previous concentration. Upon division, the cell bisects and its surface area halves.

### (n) Notes on integration and MATLAB scripts

We implemented the model using Matlab (vR2015b, The Mathworks, MA). We use a single-step forward Euler solver, with a step size of 60 s. Using a step size one order of magnitude smaller was not found to affect results substantially. The Matlab scripts are available as a repository on Github (https://github.com/twestWTCN/West_2017_PTRSB). Initial values for all parameters are shown in electronic supplementary material, table S1.
