# Supplementary material for: The origin of heredity in protocells
Source: Philos Trans R Soc Lond B Biol Sci. 2017 Oct 23;372(1735):20160419. doi: 10.1098/rstb.2016.0419 (PMC5665807; doi:10.1098/rstb.2016.0419)
Supplement: Supplementary Table 1 [file rstb20160419supp1.pdf]

The origin of heredity in protocells

Timothy West[1,2], Victor Sojo[1,2,3], Andrew Pomiankowski[1,2] and Nick Lane[1,2]

[1]*Department of Genetics, Evolution and Environment, and [2] Centre for Computation, Mathematics and Physics in the Life Sciences and Experimental Biology (CoMPLEX), University College London Gower Street, London WC1E 6BT*

[3]*Systems Biophysics, Faculty of Physics, Ludwig-Maximilian University of Munich. Amalienstr. 54. 80799 Munich, Germany.*

SI Table 1

**Initial Values**			
Symbol	***Name***	***Value***	***Notes***
$\bar{V}_{\mathrm{crys}}^{\mathrm{cyto}}(1)$	Initial mean crystal volume	1x10^{-15} cm^3	Cuboid nanocrystal of length ~500nm
$[crys]^{\mathrm{mem}}(1)$	Initial concentration of crystal in membrane	1x10^{-9} mol dm^{-3}	Set very low
$[aa]^{\mathrm{cyto}}(1)$	Initial concentration of amino acid in the cytosol	1x10^{-6} mol dm^{-3}	Set low
$[fa]^{\mathrm{cyto}}(1)$	Initial concentration of fatty acid in the membrane	1x10^{-6} mol dm^{-3}	Set low
$SA^{\mathrm{cyto}}(1)$	Initial cytoplasm surface area	4.84x10^{-6} cm^2	
$[crys]^{\mathrm{cyto}}(1)$	Initial concentration of crystal in cytosol	1x10^{-6} mol dm^{-3}	Typical particulate FeS found in hydrothermal vent samples [1]
$\boldsymbol{V}_{\mathrm{crys}}^{\mathrm{cyto}}(eq)$	Total crystal population volume (fixed)	6x10^{-10} cm^3	
**Transport**			
Symbol	***Name***	***Value***	***Notes***
$P_{\mathrm{aa}}^{\mathrm{cyto}}$	Permeability coefficient for amino acids from cytosol	1x10^{-9} cm s^{-1}	Set very low
$P_{\mathrm{fa}}^{\mathrm{cyto}}$	Permeability coefficient for fatty acids from cytosol	1x10^{-9} cm s^{-1}	Set very low
$P_{\mathrm{crys}}^{\mathrm{cyto}}$	Association constant for crystal permeation from cytosol	1x10^{-12} cm s^{-1}	Set very low
$P_{\mathrm{crys}}^{\mathrm{mem}}$	Association constant for crystal permeation from membrane	1x10^{-12} cm s^{-1}	Set very low
$P_{\mathrm{crys}}^{\mathrm{surf}}$	Permeability coefficient for FeS diffusion to crystal surface	1x10^{-12} cm s^{-1}	

Crystal Growth			
Symbol	***Name***	***Value***	***Notes***
k^{grow}	Rate constant for crystal growth	$1\text{x}10^{-6}$ s^{-1}	
$\bar{V}_{\text{crys}}^{\text{min}}$	Minimum crystal size	$1\text{x}10^{-16}$ cm^3	Minimum nanocrystal length ~50nm
K_{crys}	Saturation constant of FeS crystal nucleation	$1\text{x}10^{-8}$ mol dm^{-3}	
**Catalysis and Amino Acid Interactions**			
Symbol	***Name***	***Value***	***Notes***
$[CO_2]_{\text{in}}$	Concentration of aqueous CO_2 in cytosol	$1\text{x}10^{-3}$ mol dm^{-3}	10x CO_2 concentration at Lost City hydrothermal field [2]
K_{CO_2}	CO_2 binding constant for iron-sulphur catalyst	$3\text{x}10^{-4}$ mol dm^{-3}	~0.3mM affinity of CO_2 for ferredoxins [3]
K_{aa}	Binding constant of amino acids for crystals	1 x $10^{-4.5}$-10^{-2} mol dm^{-3}	(varied in simulations)
λ_{aa}	Fraction of organic yield that is amino acid	1/10	
λ_{fa}	Fraction of organic yield that is fatty acid	1/4	
R_{cat}	Organic turnover rate per unit area	1 x $10^{-11.8}$-$10^{-9.3}$ mol cm^{-2} s^{-1}	(varied in simulations)
**Cell Geometry**			
Symbol	***Name***	***Value***	***Notes***
r^{mem}	Thickness of fatty acid bilayer	1 x 10^{-6} cm	~10nm thick bilayer in yeast [4]
ϕ_{fa}	Headgroup area of fatty acid	2 x 10^{-15} cm^2	~0.2nm^2 surface area of arachidic acid [5]
V^{cyto}	Volume of protocell cytosol	1 x 10^{-9} cm^3	Cell of ~6000 μm^3
**Concentrations**			
Symbol	***Name***	***Value***	***Notes***
$[aa]^{\text{sink}}$	Concentration of amino acids in sink	1 x 10^{-6} mol dm^{-3}	~1uM concentrations in hydrothermal fluids and plume at Lost City [6]
$[fa]^{\text{sink}}$	Concentration of fatty acids in sink	1 x 10^{-6} mol dm^{-3}	
**Constants**			
Symbol	***Name***	***Value***	***Notes***
A_{N}	*Avogadro's number*	6.023 x 10^{23} mol^{-1}	

References for SI Table 1

1. Harmandas NG, Koutsoukos PG. 1996 The formation of iron(II) sulfides in aqueous solutions. *J Cryst. Growth* **167,** 719–724.
2. Fitzsimmons JN, Boyle EA, Jenkins WJ. 2014 Distal transport of dissolved hydrothermal iron in the deep South Pacific Ocean. *Proc. Natl. Acad. Sci. USA.* **111,** 16654–61.
3. Thauer RK, Käufer B, Fuchs G. 1975 The active species of "CO2" utilized by reduced ferredoxin:CO2 oxidoreductase from Clostridium pasteurianum. *Europ. J. Biochem.* **55,** 111–7.
4. Schneiter R, Brügger B, Sandhoff R, Zellnig G, Leber A, Lampl M, et al. 1999. Electrospray ionization tandem mass spectrometry (ESI-MS/MS) analysis of the lipid molecular species composition of yeast subcellular membranes reveals acyl chain-based sorting/remodeling of distinct molecular species en route to the plasma membrane. *J. Cell Biol.* **146,** 741–54.
5. Johann R, Brezesinski G, Vollhardt D, Möhwald H. 2001 The effect of headgroup interactions on structure and morphology of arachidic acid monolayers. *J. Phys. Chem.* **105**, 2957–2965.
6. Fuchida S, Mizuno Y, Masuda H, Toki T, Makita H. 2014 Concentrations and distributions of amino acids in black and white smoker fluids at temperatures over 200°C. *Organic Geochemistry* **66**, 98–106.
